# Diagnostic accuracy of contrast-enhanced ultrasound in the detection of small renal masses: A protocol of systematic review and meta-analysis

**DOI:** 10.1097/MD.0000000000021262

**Published:** 2020-07-17

**Authors:** Jiang-feng Wu, Chao Wu, Yun-lai Wang, Zheng-ping Wang

**Affiliations:** Department of Ultrasound, Dongyang People's Hospital, Dongyang, Zhejiang, China.

**Keywords:** contrast-enhanced ultrasound, diagnostic accuracy, meta-analysis, small renal masses, systematic review

## Abstract

**Background:**

The small renal masses (SRMs) were defined that the diameter of renal masses measured by enhanced image was ≤4 cm. The diagnostic accuracy of contrast-enhanced ultrasound (CEUS) for SRMs is apparently variable among previous studies. Hence, this study will evaluate the diagnostic accuracy of CEUS in the identification of benign and malignant SRMs.

**Methods:**

A comprehensive search using the databases of Cochrane Library, Embase, PubMed, WANGFANG, and China National Knowledge Infrastructure will be carried out to identify studies in which patients with SRMs are assessed by CEUS. Two investigators will independently screen the literature and extract the data. Any discrepancies will be resolved via discussion with the senior author. Study quality will be assessed by the Quality Assessment of Diagnostic Accuracy Studies 2 tool, and pooled sensitivity and specificity of various CEUS findings for the diagnosis of SRMs will be determined. Summary receiver operating characteristic curve will be used to assess the overall performance of CEUS.

**Results:**

This study will evaluate the diagnostic accuracy of CEUS for the diagnosis of SRMs through sensitivity, specificity, positive and negative likelihood ratio, and diagnostic odds ratio.

**Conclusion:**

This study will summarize the most recent evidence that focusing on the diagnosis of CEUS for SRMs.

**Study registration:**

INPLASY202060040.

## 1 Introduction

The small renal masses (SRMs) were defined that the diameter of renal masses measured by enhanced image was ≤4 cm.^[1–3]^ In the last decades, the incidence of renal cancer was increasing by 2% every year in South America and European.^[4]^ Most of the malignant SRMs are at T1a stage.^[5,6]^ Therefore, early diagnosis and treatment are exceedingly significant for patients with SRMs to obtain a well prognosis.^[7,8]^ Conventional ultrasound, computed tomography, and magnetic resonance imaging have been widely used to evaluate SRMs.^[9–12]^

Contrast-enhanced ultrasound (CEUS) is increasing being considered as a noval modality in the diagnosis of SMRs.^[13–19]^ However, there are still inconsistent findings, and no systematic review has specifically assessed this issue. Hence, we will perform a systematic review and meta-analysis to synthesize the diagnostic accuracy of CEUS for SRMs.

## 2 Methods

### 2.1 Objective

This study aims to evaluate the diagnostic accuracy of CEUS in the diagnosis of SRMs.

### 2.2 Study registration

We have registered this study on INPLASY202060040. This meta-analysis will be conducted according to the Preferred Reporting Items for Systematic Reviews and Meta-Analyses (PRISMA) guidelines, which include 27 items and provide specific guidance for reporting of systematic reviews.^[20]^

### 2.3 Eligible criteria for including studies

#### 2.3.1 Type of studies

Randomised control trials and case control or prospective studies will be included.

#### 2.3.2 Type of participants

Studies involving patients with SRMs will be included.

#### 2.3.3 Type of index test

Index test: Studies using CEUS for the diagnosis of SRMs will be included.

Reference test: Studies using reference standards such as histopathology, cytopathology, and/or clinical follow-up will be included.

#### 2.3.4 Type of outcome measurements

The primary outcomes are sensitivity and specificity. The secondary outcomes are positive likelihood ratio, negative likelihood ratio, and diagnostic odds ratio.

### 2.4 Information sources and search strategy

#### 2.4.1 Electronic searches

Cochrane Library, Embase, PubMed, WANGFANG, and China National Knowledge Infrastructure will be systematically searched to identify potentially eligible studies from inception to May 2020. Computer searches will be carried out using the Medical Subject Heading and keywords. Search strategy for PubMed is presented in Table 1. Similar search strategies will be adapted to other electronic databases. There will be no limitations of language and publication status.

**Table 1:**
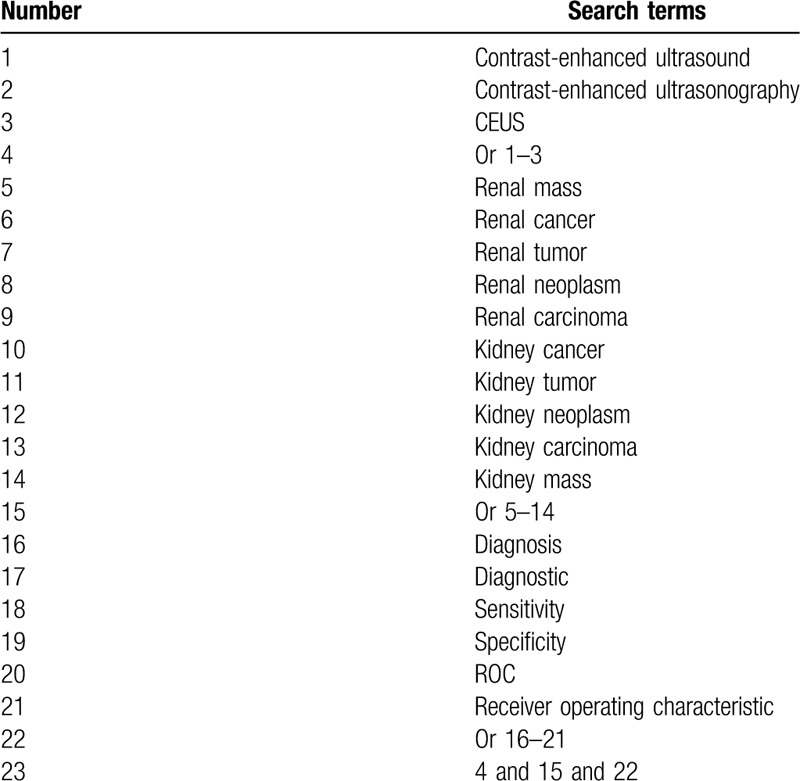
Search strategy applied in PubMed.

#### 2.4.2 Other resources

The bibliographies of identified studies and review articles will be manually screened to expand the number of eligible studies.

### 2.5 Data records and analysis

#### 2.5.1 Selection process of studies

We will export all articles from the searched results to the Endnote 7.0, and any duplicated studies will be removed. Two investigators will independently screen all literature to check whether they meet the specific inclusion criteria, and all irrelevant studies will be excluded. Then, full-text articles that meet the specific inclusion criteria will be obtained and judged. The whole process of study selection will be shown in a flowchart. Any divergences between the 2 investigators will be solved via discussion with a senior author when necessary. A list of excluded reasons alongside the rationale of their exclusion will be noted in an additional file.

#### 2.5.2 Data collection and management

Two researchers will independently extract the relevant data from the included studies using a predesigned data collection form. Any discrepancies will be resolved via discussion with the senior author. For eligible studies, the following items will be extracted: last name of the first author, year of publication, country, study type, blinding method, US equipment, probe frequency, sample size, race, mean age, gender, US diagnostic criteria, standard reference, lesion length, time between CEUS and the standard reference, true positives, true negatives, as well as false positives and false negatives of CEUS in the diagnosis of SRMs. If insufficient information occurs during the period of data collection, we will contact corresponding authors to obtain it.

### 2.6 Study quality assessment

The Quality Assessment of Diagnostic Accuracy Studies-2 (QUADAS-2) tool will be utilized to evaluate the risk of bias and methodological quality by 2 investigators independently.^[21]^ Any discrepancies will be resolved via discussion with a senior author. The quality of each included study will be evaluated by an appraisal of the risk of bias of four domains and clinical applicability of three domains of the study characteristics. Four domains consisted of patient selection, index test, reference standard and flow, and timing. Each domain will be evaluated for risk of bias, and the first 3 domains will be evaluated for applicability. The processing of the quality assessment will be performed utilizing RevMan 5.3 software (Nordic Cochrane Centre, Copenhagen, Denmark).

### 2.7 Statistical analysis

The present meta-analysis will be conducted by Stata 12.0 (Stata Corporation, College Station, TX). All statistical analyses will be performed by one investigator, who has experience in performing meta-analysis. The summary estimates of sensitivity, specificity, positive likelihood ratio, negative likelihood ratio, and diagnostic odds ratio with corresponding 95% confidence intervals will be calculated using a bivariate random effect model in the present analysis, which indicate the accuracy of CEUS in the diagnosis of SRMs. Meanwhile, the summary receiver operator curve will be constructed and the area under the curve (AUC) will be calculated. An AUC close to 0.5 shows a poor test, while an AUC of 1.0 demonstrates a excellent diagnostic test.^[22]^ We will be apply the spearman correlation analysis to determine whether a threshold effect is present, with *P* < .05 representing a threshold effect. The Cochrane *Q* test and the inconsistency index (*I*^2^) will be used to assess the heterogeneity among different studies with a *P*-value <.1 or *I*^2^ > 50% considered significant for heterogeneity.^[23]^ Meta-regression analyses utilizing several covariates will be carried out to investigate the potential causes of heterogeneity.

### 2.8 Additional analysis

#### 2.8.1 Subgroup analysis

We will perform a subgroup analysis based on the characteristics of different studies or patients, comparators, and outcomes.

#### 2.8.2 Sensitivity analysis

We will plan to conduct a sensitivity analysis by removing low quality studies to check the robustness of outcome results.

#### 2.8.3 Reporting bias

We will check reporting bias using funnel plots and associated regression tests if necessary.^[24]^

### 2.9 Ethics and dissemination

This study does not need ethical approval because it will not analyze individual patient data. The results of this study will be submitted on a peer-reviewed journal.

## 3 Discussion

We will systematically and comprehensively search more electronic databases and other literature sources to avoid missing potential studies. Two independent investigators will conduct study selection, data extraction and study quality assessment. Any discrepancies will be resolved via discussion with the senior author. The study quality will be evaluated by using QUADAS-2 tool. Prior studies assessing the accuracy of CEUS in the diagnosis of SRMs have been published, with variable sensitivity and specificity.

To our knowledge, no studies have comprehensively evaluated the literature on SRMs diagnosis by using CEUS. Hence, we will carry out a systematic review and meta-analysis to synthesize the diagnostic accuracy of CEUS for SRMs.

## Author contributions

**Conceptualization:** Jiang-feng Wu, Yun-lai Wang, Chao Wu, Zheng-ping Wang.

**Data curation:** Jiang-feng Wu, Yun-lai Wang.

**Formal analysis:** Jiang-feng Wu, Yun-lai Wang, Zheng-ping Wang.

**Funding acquisition:** Zheng-ping Wang.

**Investigation:** Jiang-feng Wu.

**Methodology:** Jiang-feng Wu, Yun-lai Wang, Zheng-ping Wang.

**Project administration:** Zheng-ping Wang.

**Resources:** Zheng-ping Wang.

**Software:** Jiang-feng Wu, Yun-lai Wang.

**Supervision:** Zheng-ping Wang, Chao Wu.

**Validation:** Jiang-feng Wu, Yun-lai Wang, Zheng-ping Wang.

**Visualization:** Yun-lai Wang, Zheng-ping Wang.

**Writing – original draft:** Jiang-feng Wu, Yun-lai Wang, Zheng-ping Wang.

**Writing – review & editing:** Jiang-feng Wu, Yun-lai Wang, Zheng-ping Wang.
